# ‘Statistical Irreproducibility’ Does Not Improve with Larger Sample Size: How to Quantify and Address Disease Data Multimodality in Human and Animal Research

**DOI:** 10.3390/jpm11030234

**Published:** 2021-03-23

**Authors:** Abigail R. Basson, Fabio Cominelli, Alexander Rodriguez-Palacios

**Affiliations:** 1 Division of Gastroenterology and Liver Diseases, Case Western Reserve University School of Medicine, Cleveland, OH 44106, USA; axb860@case.edu (A.R.B.); Fabio.Cominelli@uhhospitals.org (F.C.); 2 Digestive Health Research Institute, University Hospitals Cleveland Medical Center, Cleveland, OH 44106, USA; 3 Mouse Models, Silvio O’Conte Cleveland Digestive Diseases Research Core Center, Cleveland, OH 44106, USA; 4 Germ-Free and Gut Microbiome Core, Digestive Health Research Institute, Case Western Reserve University, Cleveland, OH 44106, USA

**Keywords:** violin plots, random sampling, analytical reproducibility, microbiome, fecal matter transplantation, data disease subtypes, personalized medicine, maltodextrin, dip test

## Abstract

Poor study reproducibility is a concern in translational research. As a solution, it is recommended to increase sample size (N), i.e., add more subjects to experiments. The goal of this study was to examine/visualize data multimodality (data with >1 data peak/mode) as cause of study irreproducibility. To emulate the repetition of studies and random sampling of study subjects, we first used various simulation methods of random number generation based on preclinical published disease outcome data from human gut microbiota-transplantation rodent studies (e.g., intestinal inflammation and univariate/continuous). We first used unimodal distributions (one-mode, Gaussian, and binomial) to generate random numbers. We showed that increasing N does not reproducibly identify statistical differences when group comparisons are repeatedly simulated. We then used multimodal distributions (>1-modes and Markov chain Monte Carlo methods of random sampling) to simulate similar multimodal datasets A and B (*t*-test-*p* = 0.95; N = 100,000), and confirmed that increasing N does not improve the ‘reproducibility of statistical results or direction of the effects’. Data visualization with violin plots of categorical random data simulations with five-integer categories/five-groups illustrated how multimodality leads to irreproducibility. Re-analysis of data from a human clinical trial that used maltodextrin as dietary placebo illustrated multimodal responses between human groups, and after placebo consumption. In conclusion, increasing N does not necessarily ensure reproducible statistical findings across repeated simulations due to randomness and multimodality. Herein, we clarify how to quantify, visualize and address disease data multimodality in research. Data visualization could facilitate study designs focused on disease subtypes/modes to help understand person–person differences and personalized medicine.

## 1. Introduction

Multimodal diseases are those in which affected subjects can be divided into subtypes; for instance, “mild” vs. “severe” disease, based on (known/unknown) modifiers of disease severity. Data subtypes, also known as “data modes”, can be visualized as “peaks” and “valleys” within a violin or Kernel plot. There is emerging interest in understanding dataset multimodality and identifying strategies to address such source of variability in disease and medical research (brain [1,2], biobanking [3], genomics [4,5], and orthopedics [6]). In animals, for example those that receive human gut/fecal microbiota transplantations (hGM-FMT) or animals administered special diets or treatments may also exhibit “high”, “middle”, or “low reactivity” (e.g., gut inflammation) in response to the intervention. Although high/low ranging responses often appear in study datasets (biological and nonbiological) as multimodal distributions, little is known about how these could affect rodent research reproducibility or how to address such multimodal and random variance. Herein, we illustrate how multimodality via random sampling affects study reproducibility in research using, as an example, fecal microbiota transplantation studies in rodents as a way to exemplify variability and randomness resulting from data multimodality

To establish the causal connection between human diseases and the microbiome, animal models, primarily germ-free models transplanted with hGM, have been widely used as tools in translational research. Unfortunately, despite efforts to help scientists improve their studies (e.g., ARRIVE guidelines), there are still concerns on poor study reproducibility, in part owing to microbiome variability [7,8]. Novel sources of artificial microbiome heterogeneity that could explain variable hGM study results have been described [8,9,10,11,12]. Recently, we also illustrated how scientists often lack appropriate methods for the analysis of cage-clustered data, and with examples, we showed how to use study power (*p* = 1 − β) to help investigators monitor their study validity and sample size (N) [8].

With respect to N, published recommendations often include to increase N, i.e., adding more subjects (e.g., human, mice, cells) to improve research reproducibility. The objective of our study was to illustrate via simulations (using as an example hGM rodent disease data dispersion/variability) the impact of repeated random sampling from a population of subjects (at various N) on (i) the data distribution, (ii) the shape of said data distribution, and (iii) the cumulative probability of generating a statistically significant result for simulated repeated hGM-transplanted group comparisons for a hypothetical disease outcome. By using various methods of random number generation, encompassing unimodal and multimodal distributions, we illustrate that randomness alone introduces large-scale ‘random analytical–statistical irreproducibility’ patterns, regardless of number type (continuous or integer/categorical), especially for multimodal data distributions.

After examining the statistical content of 38 high-quality studies [13,14,15,16,17,18,19,20,21,22,23,24,25,26,27,28,29,30,31,32,33,34,35,36,37,38,39,40,41,42,43,44,45,46,47,48,49,50] assessed in a recent systematic review [51], herein, we found that scientists who increased N, concurrently reduced the number of mice/donor (MxD), indicating that statistically, scientists replace the disease variance in mice by the disease variance in humans in their hGM-FMT studies. Furthermore, supporting our previous report [8,52], studies lacked proper statistics methods to control for animal density, and most importantly with respect to data modality, we found that none of the studies considered data multimodality/violin plots. Herein, we clarify how to visualize, quantify and address disease data multimodality in human and animal research. 

## 2. Materials and Methods

### 2.1. Overall Approach

To verify our hypothesis using, as an example, the context of hGM rodent studies and N, we used published (observed) preclinical rodent univariate data (e.g., intestinal inflammation) to make simulations with randomly generated numbers to then conduct repeated standard statistical and visualization analyses. Simulations were conducted using (i) integer data that could represent, for instance, categories of disease severity varying on scoring scale systems made of positive whole numbers categories (categorical), names (nominal), or orders (ordinal data), and (ii) continuous data that could represent, for instance, body weight or inflammation severity outcomes given in positive decimal numbers, or transmembrane electric resistance which oscillate around zero between negative and positive decimal numbers. Across multiple scenarios (details below), we used various number generator software and methods encompassing at least three major statistical probability distribution classes. The first, having no data modes with equal probability of sampling numbers across a min and max range bounds (uniform, rectangle shape); the second, having one data mode where the probability of sampling a number is higher when it is closer to the center of the data set (mean) and decreases away from the center (Gaussian unimodal and bell shape); and the third, having at least two data modes where the probability of sampling a number resulted from the combined joint probabilities of at least two Gaussian probability distributions interconnected using Markov chain principles of sampling dependency (mixed Gaussian Markov chain, multimodal, and partly overlapping bell shapes). In doing so, we generated a wide array of dataset possibilities, with varying N (from 3 to 100,000/group), which we then compared statistically as treatment/subject groups using standard tests (*t*-test, or ANOVA; see Methods and sections below for justification and nonparametric alternatives). Therein, we monitored and quantified the extent to which data analysis reproducibility was influenced by randomness alone during the sampling of subjects from hypothetical populations within varying N, as well as dataset shapes numerically restricted nonarbitrarily around published means ± SD, or upper and lower values. Lastly, we used random arbitrary range parameters for additional validation.

### 2.2. Published Preclinical hGM-FMT Rodent Data Used for Simulations

To facilitate the visualization of how random sampling and disease variability influence study conclusions (significant vs. nonsignificant *p*-values) in the context of N, we conducted a series of simulations based on existing statistical methods (see simulations described below), using, as an example, preclinical hGM-FMT disease phenotyping data estimates from our own IBD studies (Basson et al.) [52] and that of Baxter et al. [21] (a study listed in a recent systematic review [7]). In brief, by transplanting feces from inflammatory bowel disease (IBD), namely Crohn’s disease (“Dis1”) and ulcerative colitis (“Dis2”), and “Healthy” donors (n = 3 donors for each “disease/healthy” state) into a germ-free spontaneous mouse model of cobblestone/ileal Crohn’s disease (SAMP1/YitFc) [52,53], Basson et al. [52] observed with ~90% engraftment of human microbial taxa after 60 days, that the hGM-FMT effect on mouse IBD-phenotype was independent of the disease state of the donor. Specifically, samples from some IBD patients and some healthy donors did not affect the severity of intestinal inflammation in mice, while the remaining donors exacerbated inflammation, indicating the presence of disease data multimodality in animal models. Comparably, Baxter et al. [17] found that differences in the number of tumors resulting in a hGM-FMT mouse model of chemically induced colorectal cancer (CRC) were independent of the cancer status of the human donors (n = 3 colorectal cancer, n = 3 healthy individuals).

In addition to published parameters, actual data points inferred from published plots, or the dataset itself were used to define the data distribution using histograms and normality density plots using Wessa.net [54]. Inspection of the distribution of observed experimental data was performed using Excel or R software, as described in [55], which uses R code, as described in [56]. To further assist in the examination of which distribution fit the data best, the R-interface implementation of the Tukey Lambda PPCC plot was used to distinguish normal, u shape, uniform, Cauchy, and logistic distributions, as described in [54,57], using R code based on [58]. To identify the best fitting distribution function that the observed data has, we used the Excel functions (TRENDLINE and Equation) and examined the R^2^ for the linear, exponential, and logarithm function (unimodal distributions) or used polynomial functions with two or more terms to describe the shape of the data distribution. Each term approximately corresponds to a mode/peak in the dataset. Model fitting used the same interface as that used for model fitting of a normal distribution to observed data, as described by [59], which used R code as described in [56].

For clarity, the purpose of this study was to illustrate the effect of randomness as an analytical component in preclinical research datasets and not to examine the validity of rodents as models of human diseases. As such, we used simulated data generated within the data distribution parameters of published data or used completely random number sets drawn from various distributions within arbitrary number ranges, e.g., common to gut inflammation scores in rodents. Factors such as batch effect, gender, and cage density, among others, were not considered in the simulations, because the main objective was to examine ‘random sampling’ and because such factors are not often reported in rodent publications or are inherently part of the data distribution of the published datasets [8].

### 2.3. Simulation of Hypothetical Disease Outcome Sets Using Random Numbers

Iteration of random number generation [60] was conducted to illustrate the effect of random sampling on the reproducibility of analysis of mouse preclinical datasets generated using established software. In sequence, we first defined and used the published (observed) rodent disease outcome parameters when available (e.g., mean and SD, or the min and max data ranges, for at least two “subject/treatment groups”). We then used such parameters as input for random generation software (Excel, GraphPad, R software, wessa.net, and random.org), which for each iteration, generated sets of “randomly sampled treatment groups” of random numbers, which were then statistically compared using standard methods and software to determine (i) to what extent the differences were significant, (ii) the difference in magnitude between the compared groups (treatment effect difference), and (iii) which group was higher mean (direction of the effect). Each set of random numbers (a subject/treatment group) used for statistical analysis was generated at various sample sizes N to examine the effect of N on statistical reproducibility for the simulated published datasets. Results were monitored manually for each iteration and plotted to illustrate effects in manuscript figures, recorded using Excel functions by creating an analysis template simulator for readers use, or used Monte Carlo simulations in statistical software (GraphPad, or R) to compute thousands of iterations and summarize the statistical results for cumulative reproducibility and compute Monte Carlo adjusted *p*-values. 

### 2.4. Group Simulations Using Pseudorandom Integer and Continuous Numbers

To enable the visualization of the simulation strategy and analytical comparisons across integers and continuous data over various Ns, and to visualize the impact of adding three subjects to each group for each statistical simulation, we used Excel with the embedded formulas and functions (see Supplementary File 1). The supplementary file contains two spreadsheets. One sheet shows the layout for the generation of random integer numbers in increments of 3, as well as the cumulative statistics using *t*-test functions to compare pairs of data with N ranging from 3 to 63, expandable to ~1 million rows, and for uniform and Gaussian (based on inverse Gaussian functions as described below). The other sheet follows the same format, based on the same distributions, but it generates continuous random numbers, instead of integers. Pre-set bar plots with standard deviations and line plots with the cumulative summary of statistical results illustrate there is no difference between uniform or Gaussian distribution-based simulations. To allow for reproducibility, statistical analyses were completed with a suited two-group parametric (*t* test) statistical functions available in Excel, because corresponding nonparametric tests are not available in the software, and their performance is similar to parametric in numerous scenarios, especially with large N. Nonparametric statistical functions are available for Excel using third-party open-access macros and extensions that vary in implementation across platforms (e.g., Real Statistics Using Excel [61]).

As laid out in Supplementary File 1, random numbers were generated using uniform distributions, which is the standard function for Excel RAND (continuous) and RANDBETWEEN (integer) functions. However, for the generation of numbers, based on a Gaussian distribution (not readily available in Excel), we nested the RAND formula inside of the NORMINV formula for the probability input, which, in turn, returns the inverse of the normal cumulative distribution for the specified mean and standard deviation. Additional options available in Excel were not used in this study. To constrain the data range within positive numbers, since inflammation scores are not negative, we used the formula = MIN (MAX(NORM.INV(RAND(), C$16, C$17), 0),80) to limit numbers between 0 and 80, which is beyond the absolute probability of 1 of having the maximum possible inflammatory score within the expectations of the published parameters (i.e., maximum inflammation is unlikely to be 80) [62,63].

### 2.5. Visualization of Randomly Generated Numbers

In all depicted illustrations, the randomly generated numbers used computer-software/automated-pseudorandom (seeded and unseeded) methods [64,65]. Unless described otherwise, the numbers generated (generated using uniform and Gaussian distributions) were restricted to be confined within biologically meaningful data boundaries based on published data (for example, 0 as minimum for normal histological score or intestinal inflammation and 80 as arbitrary ~3-fold the maximum possible histological score) as described. For illustration purposes, the x- or y-axes in plots were generically labeled as outcome disease severity. Simulating a situation where a scientist would recruit a trio of donors (three donors) per group at a time and was interested in conducting interim statistical analysis following the addition of every trio of donors to the study, we summarized the pairwise group analysis for the simulated disease comparisons, for various N, and for consecutively added donors as an aggregate “cumulative probability of being a significant simulation” statistic. Comparisons were deemed significant if at least one *p*-value was <0.05 across simulations.

### 2.6. Parametric vs. Nonparametric Group Statistics and Monte Carlo p-Value Estimates

Because parametric and nonparametric statistical methods often produce interchangeably/similar *p*-values, especially when data have normal distribution, and also as the group sample size N increases, as previously described, herein, for illustrative purposes, we used, unless otherwise described, primarily parametric tests to conduct the statistical analysis because in most cases N was larger than 3–6, with simulations conducted with N = 6, 9, 18, 21, and additional increments of 3 up to 63, or with N = 100, 200, 600, 1000, 10,000, or 100,000. When applicable for further validation of the data generation and specific simulations datasets in Excel, the data was exported to GraphPad, a statistical software widely used in the literature, to conduct Student’s unpaired *t*-test and/or one-way ANOVA with Tukey statistical comparisons (or the nonparametric in some scenarios with low N < 6, or as needed see below) to calculate adjusted *p*-values using Monte Carlo simulations for decimal numbers with Gaussian distribution, and to determine the % of simulations that were significant or not. For post hoc analysis, nonseeded Monte Carlo simulation function was used.

### 2.7. Markov Chain Monte Carlo Multimodal Simulations of Continuous Data

To illustrate the major role of random sampling across multiple N from multimodal data distributions, we used Markov chain Monte Carlo multimodal simulation functions and R software to obtain groups of numbers from such distributions for statistical comparisons using two-group statistics. The scripts are available in Supplementary Figure S5. Specifically, to illustrate the effect of random sampling from data simulations from multimodal distribution functions, unconstrained-parameter simulations of two mixed, yet separate, normal distributions were performed using the random walk Metropolis–Hastings algorithm [66,67], a form of dependent sampling from a proposed posterior distribution, as a well-established method of Markov chain Monte Carlo (MCMC) simulations [68], using R and STATA (v15.1). In the latter, the MCMC sampling of a new individual is dependent on the prior probability of being part of a mode within a multimodal distribution, instead of being completely random from a unimodal distribution, using a log-likelihood correction to prevent negative sigma values and also allow for asymmetrical distributions. This method is beneficial as it asymptotically converges to the true proposal distribution and so represents a more robust method of data simulation compared to other alternatives of simulating sampling from multimodal distributions (i.e., binomial and mixed normal distributions).

### 2.8. Multimodality Tests and Variability of Statistical Results

The test of multimodality was conducted using the dip test (which measures the departure of a sample from unimodality, using the uniform distribution as the worst case as a reference) and STATA [69], with packages available in R [70]. The tabulation of modes from a variable in a dataset was computed using the *modes* and *hsmode* function in STATA [71,72]. Statistical and simulation analyses were conducted or plotted with Excel, R, Stata, and GraphPad.

To determine the sources of statistical methods variability in hGM-FMT rodent studies, we reviewed the content of 38 studies listed in ref [51]. For computation purposes, we searched each article for the following keywords: “cage,” “stat*”, “housed”, “multiple”, “multivariable”, “cluster”, “mixed”, “individual*”, and “random*”, and appropriately extracted details to additional inserted columns of an excel file. Detailed statistical tests and software used, focused on assessing the effect of the hGM in the rodent phenotypes, were extracted to determine if studies used proper cluster statistical analysis and/or controlled for random effects introduced by caging, when needed, that is, if more than one mouse was housed per cage. Data including descriptions of animal density (numbers, e.g., 1–5) were assigned to the sourced keywords to allow for statistical analysis. If a range was provided for N or animal density, estimations were computed using the median value within the range, as well as the minimum and maximum values. The average of estimated center values was used for analysis and graphical summaries 

## 3. Results

### 3.1. “Disease Data Subtypes” (Modes) Occur with Uniform and Gaussian Random Sampling

**Figure 1:**
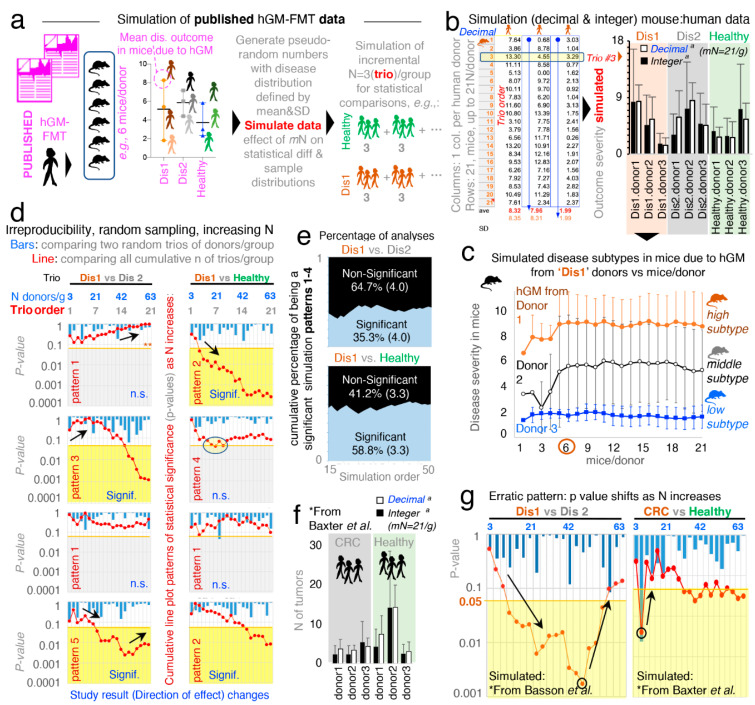
Random sampling from overlapping datasets yield unexpected “linear patterns of cumulative statistical irreproducibility”. Simulations on observed data from Basson et al. [52] to visualize disease vs. healthy datasets. (**a**) Method overview to generate pseudorandom numbers and simulations from published (observed) data (see details/formulas in Supplementary File 1 and Supplementary Figure S1). (**b**) Visualization of simulated outcomes using random decimal and integer numbers datasets generated based on 3 donors/group for Disease 1 (“Dis1”), Disease 2 (“Dis2”), and “Healthy” groups. Bar plot of N = 21 mice/donor, notice no differences between integer and decimal datasets group pattern, or absolute differences, superscript letter “a”, paired-t *p* > 0.05. (**c**) Simulation of human gut/fecal microbiota transplantations (hGM-FMT) mice data yields reproducible simulated “disease data subtypes” from 6 mice/group. (**d**) Cumulative line plots depicting patterns of statistical irreproducibility and pairwise statistical directions of effect estimates (n.s.:signif., signif.:signif., signif.:n.s., and n.s:n.s.). Representative simulations comparing two groups of donors, with N ranging from three (trio) donors/group to 63, in multiples of three (cumulative addition of new trios per group). Note *Y*-axis, *p*-value of differences using two-group Student’s *t*-test. Notice as N increases, the cumulative significance (red line) exhibit different linear patterns due to variance introduced by random sampling. (**e**) Percentage of simulated analysis with significant or nonsignificant pairwise difference (blue; significant, black; non-significant; and parentheses, SD). Comparison deemed significant, if at least one *p*-value < 0.05 across simulations with N between 3 and 63 donors/group. (**f**) Visualization of simulated outcome using observed data from Baxter et al. [21]. No differences between integer and decimal datasets, superscript letter “a”, paired-t *p* > 0.05. (**g**) Random simulations illustrate “erratic” statistical patterns. Notice as N increases, group differences become more significant, until an inflection point, where adding more donors makes the significance disappear. See Supplementary Figure S2 for additional examples and computed R^2^ value to illustrate the linearity of the correlation between N and statistical significance.

In microbiome rodent studies, the selection of a sufficient number of human donors, as well as the number of mice/group which required the testing of each human donor (MxD), is critical to account for the effects of random sampling, which exist when the hGM induces variable disease severity in humans and rodents. Thus, to visualize the variability of disease severity (data subtypes/modes) in hGM-FMT rodents and the effect of N on the reproducibility of said pairwise statistical comparisons from, hypothetically, randomly selected human donors, we first conducted a series of simulations using as input, the mean ± SD (disease outcome, continuous data) from hGM-FMT mice in Basson et al. [52] to generate random numbers (Figure 1a; note dispersed overlapping data). We also generated separate random datasets of integer and decimal numbers using functions designed to draw numbers from a uniform and Gaussian distribution (see details in Methods, and formulas and visualization strategy in Supplementary File 1 and Supplementary Figure S1). We showed that under the conditions simulated, the integer-uniform dataset is statistically similar to the one generated using decimal-Gaussian methods (Figure 1b), and we demonstrated how the random selection of N (sampled as groups for each of three iterative datasets) influences the direction and significance in pairwise comparative statistics.

Simulations showed that the number of MxD is important because mice have various response patterns to the hGM (i.e., disease severity and disease data subtypes/modes), which can be consistently detected depending on the MxD and thus the variability introduced by random sampling. Simulations showed that for the three hGM-FMT group datasets (plotted as Dis1, Dis2, and Healthy), it was possible to reproducibly identify from two to three unique donor disease severity subtypes (data modes) in mice induced by the hGM (“high”, “middle”, and “low” disease severity). 

Simulation plots made it visually evident that testing <4–5 MxD yielded mean values more likely to be affected by intrinsic variability of random sampling, thus making studies with >6 MxD more stable and preferable. Conversely, studies with 1–2 MxD are at risk of being strongly dependent on randomness. Iterative simulations showed that the mean effect (e.g., ileal histology) in transplanted mice varies minimally (i.e., stabilizes) after 7 ± 2 MxD, depending on the random dataset iterated. Beyond that, increasing MxD becomes less cost-effective/unnecessary if the focus is the human donors (Figure 1c).

### 3.2. Random Sampling from Overlapping Datasets Yield “Linear Patterns of Statistical Irreproducibility”

Often, published literature contains figures and statistical analysis conducted with three donors per disease group. Thus, to mimic this scenario and to examine the role of random sampling of subjects on the reproducibility of pairwise statistical results (significant vs. nonsignificant) in the context of hGM-FMT rodent studies, we compared two groups of donors, each having three donors/group (donor “trio”), with N increasing in multiples of three (ranging from 3 to 62 donors/group). We conducted (i) multiple donor/group (“trio–trio”) pairwise comparisons and (ii) a simultaneous overall analysis for the cumulative sum of all the donor trios (i.e., the cumulative addition of new trios per group) simulated for each disease group. That is, we monitored and quantified whether results for each random iteration (simulation event) were significant (using univariate Student’s t-statistics *p* < 0.05) or nonsignificant (*p* > 0.05) for groups of simulated donor datasets (Dis1, Dis2, or Healthy). Assessing the effect of random sampling at various N and also as N accumulated, we were able to illustrate that pairwise “trio–trio” comparisons between the simulated rodent disease outcome datasets almost always produced nonsignificant results when iterative trios were compared (due to large SD overlapping; see bars in Figure 1d representing 21 sets of pairwise trio–trio *p*-values). However, as N increases by the cumulative addition of all (mostly nonsignificant) donor trios (i.e., N increases in multiples of three, for a range of N between 3 and 63 donors/group; (3, 6, 9, 12,…,63)), pairwise statistical comparisons between the simulated datasets did not produce consistent results (see line plots in Figure 1d representing *p*-value for cumulative addition of donors when sampling iterations were simulated).

Results are clinically relevant because the simulated N, being much larger (63 donors/group) than the largest N tested by one of the hGM-FMT studies examined in a recent systematic review [51] (21 donors/group) [45] demonstrates that the analysis of randomly selected subjects would not always yield reproducible results due to the chance of sampling aleatory sets of individuals with varying degrees of disease severity, regardless of how many donors are recruited in an study. To provide a specific example, using Dis1 as a referent, cumulative pairwise comparisons (vs. Dis2 and vs. Healthy) revealed at least five different patterns of irreproducible statistical results (rodent disease outcome) as N increased between 3 and 63 per group. Figure 1d illustrates four of these variable cumulative linear patterns of statistical irreproducibility in which, remarkably, (i) Dis1 becomes significantly different vs. Dis2, and vs. Healthy, as N increases, (ii) Dis1 becomes significantly different from Dis2 but not vs. Healthy, (iii) Dis1 was significantly different from healthy but not vs. Dis2, and (iv) Dis1 never becomes significantly different despite sampling up to 63 donors/group. See Supplementary Figure S2 for complementary plots illustrating linearity of patterns (R^2^, mean 0.51 ± 0.23, 21 simulations).

Hence, the results clearly illustrate that seeking funds to recruit more donors as recently suggested is not a prudent statistical solution to the problem of understanding disease causality of widely variable conditions in both humans and animals. By statistical irreproducibility, herein, we refer to the inability to reproduce the direction and statistical significance of a test effect when analyses are conducted between groups created by the random selection of subjects from distributions defined using observed data.

To investigate the cumulative probability of generating a statistically significant simulation that collectively would lead to the inconsistent patterns (statistical irreproducibility) observed via random sampling, we computed an aggregate “cumulative probability of being a significant simulation” for 50 pairwise statistical simulation sets fulfilling the four linear patterns described above. Emphasizing the concept that increasing N is not a reproducible solution, Figure 1e shows that only 35.3 ± 4.0% of comparisons between Dis1 and Dis2, and 58.8 ± 3.3% for Dis1 and Healthy, were significant.

### 3.3. “. Erratic Patterns” of Statistical Irreproducibility as N Increases

To increase the external validity of our observations, we next simulated the data published from a hGM-FMT study on colorectal cancer conducted by Baxter et al. [21]. In agreement with Basson et al. [52], Baxter et al. revealed comparably bimodal colorectal cancer phenotypes in mice resulting from both the diseased (colorectal cancer) patients and healthy human donors (Figure 1f).

Unexpectedly, we observed for both Basson et al. [52] and Baxter et al. [21], as simulations were conducted, an “erratic” shift on the significance of the cumulative analysis occurred as N increased (Figure 1g). In some cases, the increasing addition of donor trios/group (as simulations proceeded for increasing values of N) made it possible to identify simulations where erratic changes in the statistical significance for group comparisons switched randomly, yet gradually, from being significant to nonsignificant as more donor trios were “recruited” into the simulations (Figure 1g). Clinically relevant simulations indicated that adding extra subjects could at times actually invert the overall cumulative effect of the *p*-value, possibly due to the variable distribution and multimodal nature of the host responses to experimental interventions. As such, simulations indicate that it is advisable to conduct several a priori determined interim data analysis in clinical trials to ensure that significance is numerically stable (*p* < 0.05), as well as the relevance of personalized analysis to examine disease variance in populations. Unfortunately, there are no guidelines or examples available to assist in determining how many donors would be sufficient, and to visualize the effect of random sampling of individuals from a vastly heterogeneous population of healthy and diseased subjects.

### 3.4. Monte Carlo Simulations and Probability of Statistical Reproducibility

Expanding the reproducibility of these uniform and Gaussian distributions, we then made simulations using solely Gaussian distributions for N = 63 donors/group and conducted (i) Monte Carlo adjusted Student’s unpaired *t*-tests and (ii) Monte Carlo adjusted one-way ANOVA with Tukey correction for family errors and multiple comparisons. Monte Carlo simulations were used to indicate how many tests will yield a significant result and the direction of effect. Monte Carlo simulations with normal Gaussian distribution around the group means and a pooled SD of ± 4 were also computed. See Supplementary Figure S3 and Supplementary File 2 for methods employed in GraphPad for this Monte Carlo simulation and the corresponding dataset. Supporting the observations above, Monte Carlo Gaussian simulations showed that, using pairwise comparison, Dis1 would be significantly different from Dis 2 (adjusted *t*-test *p* < 0.05) only 57.7% of the time (95% CI = 58–57.4), with 1540 simulations producing negative (contradictory) mean differences between the groups. Compared to Healthy, Dis1, and Dis2 were significant only 9.1% (95% CI = 9.2–8.9) and 78.3% (95% CI = 78.6–78.1) of the time, respectively. Statistical analyses were compared, for *p*-values computed by parametric *t*-tests and nonparametric Mann–Whitney statistics, findings were comparable, yet distinct, with borderline significant *p*-values.

Under the “Weak Law of Large Numbers” [73,74,75] and randomization principles, it is almost always possible to detect some level of statistical significance(s) and mean group differences when asymptotic mathematical methods based on numerous simulations are used. For example, when simulations are used as a surrogate for multiple experiments which are not possible in real research settings. However, in this case, the mean simulated differences yielding from 100,000 simulations were minuscule (Dis1-Dis2 = 1.6; Healthy-Dis2 = −1.97; and Healthy-Dis1 = 0.42). Compared to the range of disease variance for each disease, such minuscule differences may not be clinically relevant to explain disease variance at the individual level. Note that the SD was 4; therefore, it is intuitive to visualize in a numerical context such small differences across greatly overlapping unimodal simulations. 

Correcting for family errors, one-way ANOVA corrected with 10,000 Monte Carlo simulations with N = 63/group showed that at least one of the three groups would be statistically different in approximately only 67.2% of the simulations (95% CI = 64.2–70.0), whereas in 32.8% (95% CI = 64.2–70.0) of simulations, the groups would appear as statistically similar (see Supplementary Table S1 for estimations after 100,000 Monte Carlo simulations (R software); note narrower CI as simulations increase, Supplementary Figure S4). The comparison of Dis1 vs. Dis2 in supplementary Table S1 demonstrates that the percentage of cases in which a simulation could be significant, depending on the degree of data dispersion. For example, simulations with SD of 4, compared to SD of 10, produce significant results less often, illustrating how data with larger dispersions contribute to poor statistical reproducibility, which cannot necessarily be corrected by increasing N.

### 3.5. Violin Plots to Visualize, and Tests to Quantify, Multimodal “Disease Data Subtypes”

**Figure 2:**
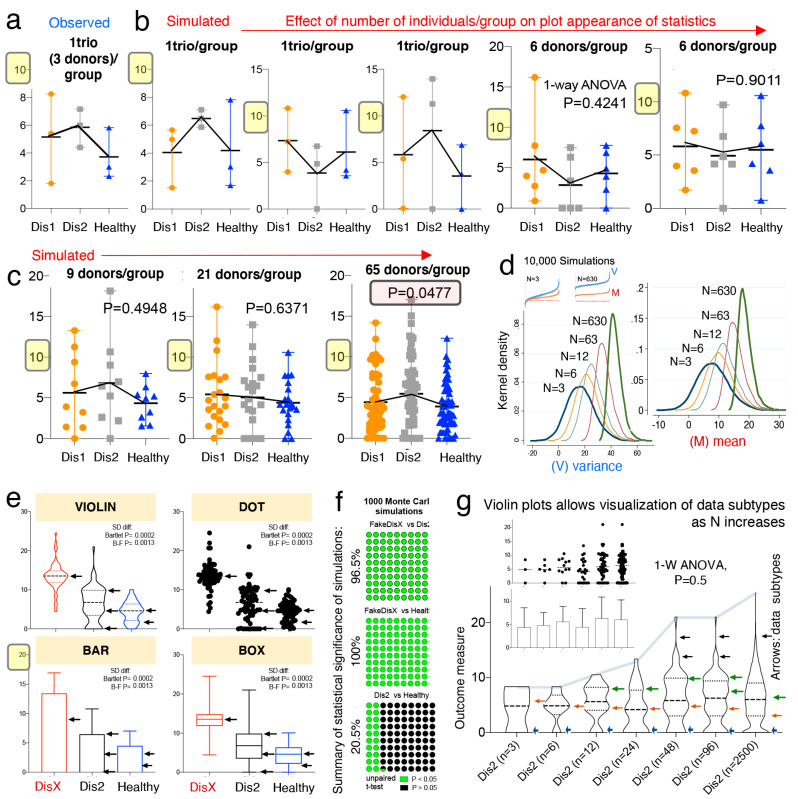
Violin plots enable visualization of data subtypes in simulations of random sampling for various N sample size. Observed raw data derived from Basson et al. (**a**,**b**) Dot plots (mean, range) of observed (1 “trio”; 3 donors/group), and simulated data (3 and 6 donors/group; panel B). Note that differences are not significant because of the variability between diseases. (**c**) Dot plots (mean, range) of simulated data for 9, 21, and 65 donors per group. Note that simulated mean effects became significant with 65 donors/group. However, the mean difference is small compared to the variance of the groups and the difference is not biologically different because it is a function of the total variance (23%). (**d**) Kernel density simulations (10,000) based on observed (n = 3) and simulated data. Note that as N increases the mean becomes narrower while the variance widens. See 100,000 Monte Carlo simulations in Supplementary Table S1. (**e**) Comparison of visual appearance and data display for violin, dot, bar, and box plots of simulated data to illustrate “disease data subtypes” (arrows). (**f**) Plot illustrates cumulative proportion of simulation runs that generated a significant (green, *p* < 0.05) or nonsignificant value (black, *p* > 0.05). Analysis illustrates how Monte Carlo adjusted analysis could be reported with observed findings. See Supplementary Figure S4 for 100,000 Monte Carlo simulations of random numbers generated in R. (**g**) Violin plots allow visualization of data subtypes as N increases (arrows, subtypes).

To visualize and to illustrate how to address the underlying mechanisms that could explain the “linear and erratic patterns of statistical irreproducibility” that is introduced by random sampling, we first used dot plots based on observed and simulated data, followed by kernel-based statistics and plots (violin, box, bar). Plot appearance and one-way ANOVA statistics showed that when N is increased, significant results, when present for largely overlapping phenotypes, are primarily due to small differences between sample means (Figure 2a,b).

Simulations that compared three groups of 65 donors/group almost always yielded a significantly different group; however, dot plots show that the significant differences between means are just a small fraction of the total disease variability as verified with Monte Carlo simulations. That is, as N increases, comparisons can become significant (see plot with 65 donors in Figure 2c). In this context, a significant difference of such a narrow magnitude may not be clinically relevant, or generalizable, to explain the presence of a disease phenotype in a population, especially for subjects at the extreme ranges of the disease distribution.

Mechanistically, the detection of significant comparisons can be attributed to the effect that increasing N has on the data mean and variance, which increases at a higher rate for the variance as shown in Figure 2d. Instead of increasing N as a general solution, we suggest that scientists use violin plots over other plots commonly encouraged by publishers [76] (e.g., bar, boxplot, and dot plots), because violin plots provide an informative approach to make inferences about “disease data subtypes” in the population (see subtypes shown with arrows in Figure 2e,f).

Violin plots are similar to a box plot, as they show a marker for the data median, interquartile ranges, and the individual data points [77]. However, as a unique feature, violin plots show the probability density of the data at different values, usually smoothed by a kernel density estimator. The idea of a kernel average smoother is that within a range of aligned data points, for each data point to be smoothed (X0), there is a constant distance size (λ) of choice for the kernel window (radius or width), around which a weighted average for nearby data points are estimated. Weighted analysis gives data points that are closer to X0 higher weights within the kernel window, thus identifying areas with higher data densities (which correspond to the disease data modes). As an example of the benefits of using violin plots, Figure 2g shows that as N increases, as does the ability of scientists to subjectively infer the presence of disease subtypes. 

To strengthen the reproducibility of “subtype” mode identification, herein, we also suggest the use of statistical methods to identify disease data modes (e.g., see the statistical function *modes* in Methods and Discussion), because as N increases, the visual detection of modes becomes increasingly more subjective as shown in Figure 2g.

### 3.6. Violin Plots Guide Subtype Analysis to Identify Biologically Significant Results

**Figure 3:**
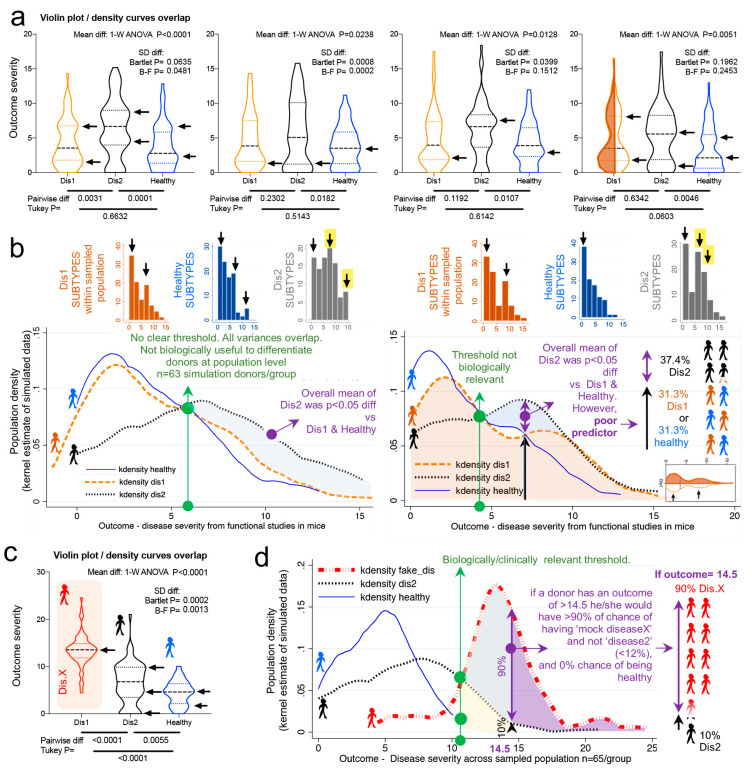
Violin plots illustrate that statistical differences with large N may not have clinical predictive value at individual level. Violin and kernel plots illustrate statistical vs. biologically relevant differences and thresholds. (**a**) Violin plots of four simulated random number sets illustrate that each set of donors may have unique subtypes of disease illustrated with arrowheads (disease severity scores with higher number of simulated donors). Arrows indicate “disease data subtypes” vary with every simulation of 63 donors/group. (**b**) Kernel density curves illustrate large overlap of sample population from simulated data (see panel 3a). Significant differences are highlighted by shaded area. Note the threshold does not have distinctive separation for the plots indicating that it is not biologically useful as a predictor of outcome. (**c**) Violin plots illustrate meaningful statistical difference for population (compared to panel 3b). “Fake disease X” (“DisX”) was generated as a “mock” disease following Gaussian distribution around the mean. Monte Carlo simulations were significant 96.5% (upper limit 97.6, lower limit 95.4%). (**d**) Kernel density curves of panel 3c illustrate example of distribution separation with both statistical difference and biological relevance.

Violin plots and kernel density distribution curves in Figure 3 illustrate why comparing groups of randomly sampled individuals may not yield biologically relevant information, even though statistical analysis identifies that the mean values differ between compared groups. Figure 3a illustrates the different patterns of potential donor subtypes (i.e., data modes visualized in violin plots as disease data/curve “peaks”) that would yield significant results in a single experiment depending on the donors sampled. 

However, the kernel density plots in Figure 3b show that significant findings do not necessarily indicate/yield clinically relevant thresholds or parameters to differentiate between the populations (due to the overlapping and inflation of data “peaks” in some subjects within the samples). To contrast the data simulated from Basson et al., we replaced data from Dis1 dataset with a Gaussian distributed (R software) sample of random numbers (within 13.5 ± 3.5, labeled as “fake disease X”; vs. 6.4 ± 4.3, and 4.5 ± 2.5 for Dis2 and Healthy, respectively) to illustrate how a kernel plot would appear when significant differences have a clinically relevant impact in differentiating subtypes (Figure 3c,d).

Collectively, simulations indicate that the uneven random sampling of subtypes across a disease group would be an important factor in determining the direction of significance if studies were repeated, owing primarily to the probability of sampling data “modes” or “peaks/valleys” in both healthy and diseased populations.

### 3.7. Multimodal Datasets Illustrate How Statistical Irreproducibility Occurs

Thus far, we have used unimodal distributions to show how random sampling affects statistical results. However, there has been an increased interest in understanding data multimodality in various biological processes [78,79] for which new statistical approaches have been proposed. Methods to simulate multimodal distributions are however not trivial, in part due to the unknown nature of multimodality in biological processes. 

**Figure 4:**
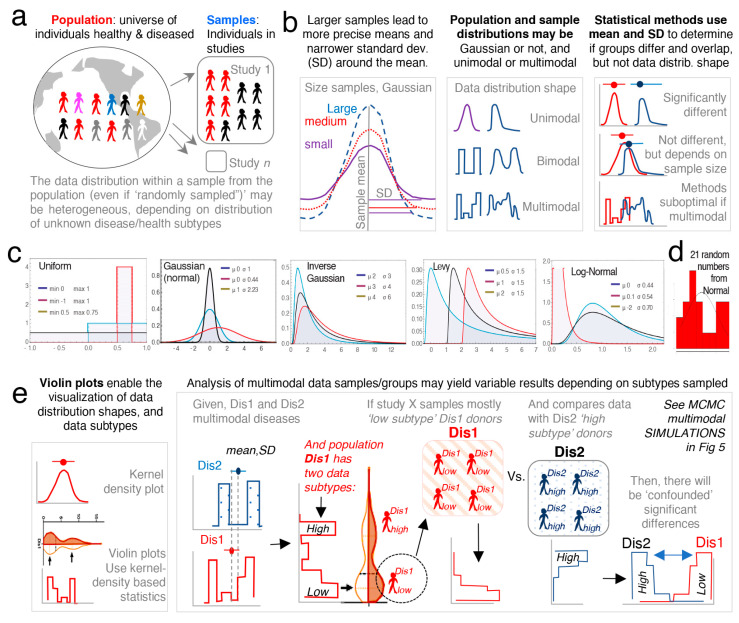
Conceptual overview of the effect of random sampling and analysis of multimodal data. (**a**) Schematic conceptualization of random sampling from a population of heterogeneous individuals. (**b**) Representation of various unidimensional data distributions. Notice that mean and SD do not represent the shape of the distribution. (**c**). Examples of probability density functions of unimodal distributions. Wolfram language [80]. (**d**) Example of a random sampling of numbers generated in this study using decimal Gaussian distribution generates a non-Gaussian distribution (bimodal; two peaks/modes, as in disease distribution subtypes). This illustrates that even under seemingly unbiased circumstances (randomness), a set of random subjects from a population may be of two subtypes and not represent the population in its “universe” of disease possibilities. (**e**) Multimodal distributions. Use of violin and kernel plots to visualize subtypes.

To facilitate the understanding of the conceptual mechanisms that influence the effect of data multimodality and random sampling on statistical significance, Figure 4a–e schematically contextualizes the statistical and data distribution principles that can interfere with reproducibility of statistical results when simulations are repeated. 

Random simulations from unimodal distributions work on the assumption that numbers (e.g., donors’ disease severity) are drawn from a population, independently from one another. That is, the probability of sampling or drawing a number from a population is not influenced by the number that was selected prior. While this form of random sampling is very useful in deterministic mathematics, it does not capture the dependence of events that occur in multimodal biology. That is, in biology, the probability of an event to occur depends on the nature of the preceding events. To increase the external validity of our report, we thus conducted simulations based on three strategies to draw density curves resembling multimodal distributions.

**Figure 5:**
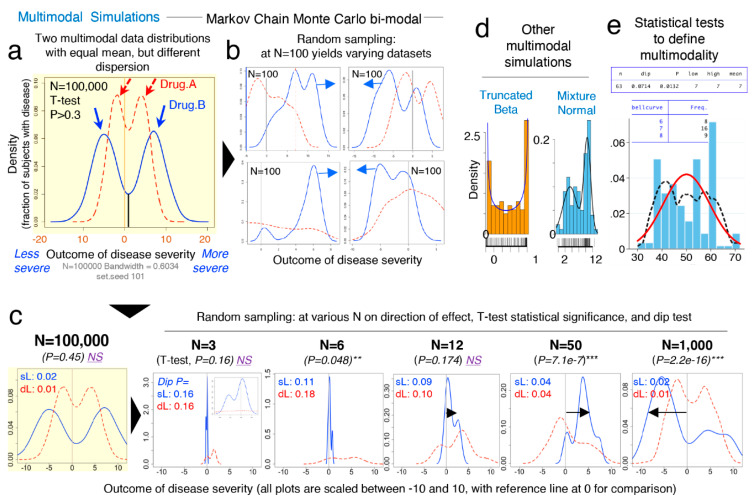
Comparison of two statistically similar multimodal datasets yields highly irreproducible results due to random sampling at various N. Markov chain Monte Carlo (MCMC) simulations emphasize the need to identify disease subtypes for the study of multimodal diseases. MCMC are random-number-generating strategies to simulate two multimodal distributions, wherein a random walk MCMC Metropolis–Hastings’ algorithm simulates random sampling accounting for inter-/within-mode dependence of two “data disease subtypes”. (**a**) MCMC simulations of two statistically similar multimodal datasets (hypothetical effect of “drug A”; red dash line vs. “drug B”; blue solid line), “real” distributions set at N = 100,000; grey reference line at x = 0, downward arrows; data modes. (**b**) Random sampling of multimodal dataset (from panel 5a) at N = 100 yields varying dataset distributions and a different mean, SD. (**c**) Effect of random sampling dataset (from panel 5a) on increasing N on *t*-test significance and direction of effect for “drug A” vs. “drug B” (arrow), including dip test for MCMC simulations (*set.seed* 101). See Supplementary Figure S5 for wider range of N and the STATA and R command scripts for the *dip test* and *modes* analyses. (**d**) Example of other multimodal distributions derived from “truncated beta” and the combination of two “mixed unimodal” distribution functions. (**e**) Example of a Hartigan–Hartigan (Hartigans’) unimodality *dip test* and a *modes* test [69,83,84] showing a multimodal data distribution (black dotted line) compared to normal univariate density plot (red line). To identify data subtypes (modes), the dip test [69] computes a *p*-value to help determine unimodal or multimodal; does not require a priori knowledge of potential multimodality; it is interpreted from test statistics (if *p* < 0.05 data is not unimodal, if *p* > 0.05–1.0 at least one data mode in dataset). Asterisks indicate significance *p* < 0.05.

To simulate the statistical comparison of two hypothetical multimodal data distribution, we (i) ran Markov chain Monte Carlo (MCMC) simulations for two datasets (“drug A” vs. “drug B”) each with two data modes (Figure 5a,b), (ii) used the statistical *dip test* (STATA) to determine if the simulated data were statistically multimodal, and (iii) used the Student’s *t*-test to determine the statistical significance, the mean differences, and directions for the simulated distributions (“drug A” vs. “drug B”), using various N (Figure 5c). The MCMC simulations clearly illustrate how random sampling of two multimodal hypothetical datasets lead to inconsistent patterns of statistical results when compared, indicating that biological data are multimodal, have multiple peaks/modes, and that two groups intended for comparison may have different or mismatching shapes and thus real data may not have Gaussian distribution. Notice that the data dispersion increases as N increases; see summary statistics in Figure 5c.

Collectively, Figure 5 underscores the notion that randomness alone elicits effect on irreproducible results, and that mean-SD are imperfect to visualize data shape. See Supplementary Figure S5 for wider range of N and the scripts for the *dip test* and *modes* analysis using STATA and R commands.

Figure 5d,e depicts distributions derived from both “truncated beta”, and the combination of two “mixed unimodal” distribution functions (e.g., two independent Gaussian curves in one plot), which are illustrative of multimodality, but not necessarily reliable methods to examine the effects from dependent random sampling in multimodality. Thus, we used “Random walk Markov chain Metropolis–Hastings algorithms” using R software to simulate random sampling, accounting for the hypothetical dependence between two different disease subtypes.

Conclusively, MCMC illustrations emphasize that increasing N in the study of multimodal diseases in a single study should not be assumed to provide results that can be directly extrapolated to the population, but rather, MCMC emphasizes that the target study of data subtypes could lead to the identification of mechanisms which could explain why diseases vary within biological systems (e.g., humans and mice). 

### 3.8. Categorical Data Exhibit Multimodality

Until this point, the majority of data simulations reported herein were based on continuous data, using various methods computer pseudorandom number algorithms. Statistical comparisons were then made between two and three groups per simulation using *t*-tests or one-way ANOVAs, or their nonparametric equivalent, as it is common in rodent literature. To further understand the effect of randomness on preclinical datasets, we further simulated categorical outcomes [81,82]. We simulated five categories of gut inflammation, with changing severity in steps of 1, between 1-(category “normal”) to 6-(category “most inflamed”). To illustrate how randomness affects the reproducibility of statistical analysis in studies with >3 treatments, we simulated five treatment groups (untreated, placebo, and treatments X, Y and Z). 

Using an integer generator which draws true random numbers from atmospheric noise (random.org), we set the algorithm to draw random numbers between 1 and 6 (representing the six categories), creating equal group sets of N = 6, 12, 100, and 1000 integers/group, with no differences between the five groups.

One-way ANOVA (and Kruskal–Wallis) statistics with post hoc pairwise comparisons across groups for >250 study dataset simulations, illustrated that increasing N from 6 to 1000/group do not prevent the occurrence of expected false significant findings (i.e., *Ho* = at least one group is different) with *p*-value < 0.05. Random groups with N = 1000 expected to be similar, showed statistical significances in 3 of 50 iterations, which is expectedly similar to the expected five false discoveries for 100 if *p* = 0.05 (3/50 vs. 5/100; Fisher exact *p* = 1); however, the directions of the effects changed drastically within treatment groups, across the significantly different simulations. 

**Figure 6:**
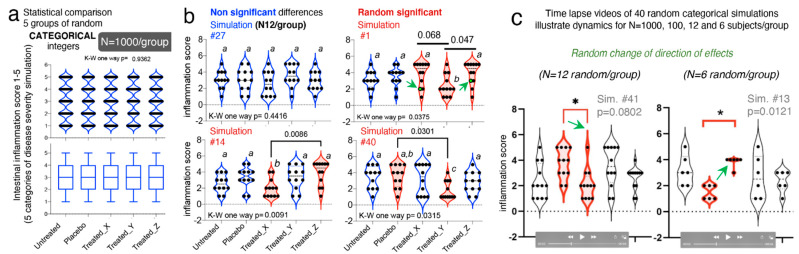
Categorical data simulations and violin plots illustrate that integer data exhibit multimodality and affect statistical reproducibility as simulations are performed randomly with various N. (**a**) N = 1000/group, simulation #1, violin plot and correspondent box plot for nonsignificant expected similarity across five groups (one-way K-W *p* > 0.05). See *p*-values and violin plots for >40 consecutive simulations in Supplementary Figure S7. (**a**,**b**) N = 12/group, violin plots for simulations #27 (non-significant), and #1, #14, and #40 (significant) illustrate comparisons due to randomness, result in irreproducible “treatment effects” and “direction of effects”. Arrows, data points with statistical influence (see boxplots in Supplementary Figure S7). (**c**) Screenshots of time-lapse videos to show dynamically how statistical irreproducibility occurs at random for various N. Arrows indicate direction of effect. See Supplementary Video S1 doi:10.6084/m9.figshare.13377407. Asterisks indicate significance *p* < 0.05.

Supporting our hypothesis, increasing N does not necessarily prevent false discoveries, as we did not see more false discoveries than linearly expected [51] when N decreased from “optimal” 1000 to “less optimal” 100, 12 and 6 (3/50 vs. 2/40, 4/40 and 1/40, respectively; Fisher exact *p* < 0.6261; Figure 6a–c).

Visualization of these integer datasets with violin plots, illustrates that in all cases, categorical data follow multimodal distribution principles that accentuate the random irreproducibility of statistical analysis, and more importantly, the irreproducibility of the direction of effect as simulations are repeated. Violin plots illustrate how with lower N, there is the risk that investigators misperceive a data point as an outlier, when it is not, and proceed to exclude such points, consciously or unconsciously favoring the appearance of significant findings, especially when using small N for categorical data. As an alternative to categorical data, we have proposed the use of decimal scoring systems, instead of univariate integers, where decimals further carry information relevant to disease severity, making the system intrinsically more multivariable (see validated examples from Rodriguez-Palacios et al. for colonoscopy scores, and pathological scores for intestinal pathologies in [53,85]). To appreciate the advantages of violin plots in understanding integer multimodality for integers, simulations at various N are available as time-lapse videos at doi:10.6084/m9.figshare.13377407 (https://figshare.com/s/dcf154ce73c5bc086e80).

### 3.9. “Data Disease Subtyping” and “Cage-Cluster” Statistics

One important caveat to consider across animal studies is that increasing N alone is unhelpful if clustered-data statistics are not used to control for animal cage-density (>1 mouse/cage), which our group showed contributes to “artificial heterogeneity”, “cyclical microbiome bias”, and false-positive/false-negative conclusions [8,86].

To infer the role of scientific decision on the need for particular statistical methods, we examined the published studies [51] for “animal density” and “statistical” content (see Methods). Supporting the need for “modernizing” data analysis, we found that only one of the 38 studies (2.6%, 95% CI = 0.1–13.8%) used proper statistical methods (mixed models) to control for cage-clustering [23].

**Figure 7:**
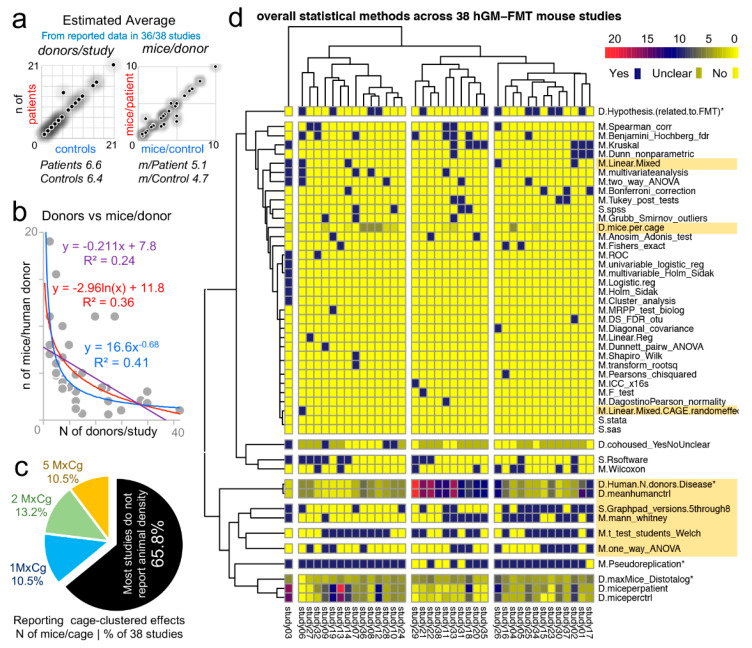
Study design and statistical methods among 38 hGM-FMT studies reveal lack of cage-clustered analysis and dominance of univariate analysis. Analysis of 38 studies reviewed in ref [51]. (**a**) Average and correlation of human donors with disease vs. healthy controls *h*N (left) and number of mice *m*N per human donor across studies (right plot). (**b**) Correlation plot with exponential, logarithmic and linear fits shows that scientists tend to use less mice when more donors are tested, creating a “trade-off” between data uncertainty due to variance in human disease with that of variance in animal models for disease of interest. (**c**) Pie chart, distribution of studies reporting mice/cage (MxCg), which indicates cage-clustered effects (search within study text keywords cage/cluster*, individual/house*, mice per*, density*, mixed/random/fix/methods/stat*, *p* = *). Most studies do not report MxCg (animal density). (**d**) Heatmap, overall statistical methods (M), statistical software (S), and study design (D) used across 38 studies. Only “study 6” [23] used linear mixed methods to control for the random effects of cage clustering. *Asterisk indicate variables examined in ref. [51]. Most statistical software reported in studies appear to be used for univariate statistics.

Although on average, studies tested 6.6 patients and 6.4 controls/group (range = 1–21), most studies were below the average (65.7%, 25/38, 95% CI = 48.6–80.4%), with 14 having <4 donors/group (Figure 7a). However, of interest, the number of human donors included in a study was inversely correlated with the number of mice/per donor used in the FMT experiments Figure 7b.

Unfortunately, the majority of studies (25/38, 65.8%, 95% CI = 48.6–80.4%) did not report animal density, consistent with previous analyses [8]; while 10.5% of the studies (4/38, 95% CI = 2.9–24.8%) housed their mice individually, which is advantageous because study designs are free of intraclass correlation coefficient, eliminating the need for cage-cluster statistics (Figure 7c).

Our review of the statistical methods used across the 38 studies also revealed that most scientists used GraphPad chiefly for graphics and univariate analysis of mouse phenotype data. This finding suggests an underutilization of the available functions in statistical software, for example, Monte Carlo simulations, to help understand the effect of random sampling on the reproducibility and significance of observed study results, and the likelihood of repeatability by others (Monte Carlo adjusted 95% confidence intervals) (Figure 7d). Of note, none of the studies considered multimodality or used violin plots (0%, 0/38, 95% CI = 6.9e−18, 9.1).

### 3.10. Multimodality in Human Outcome Data

**Figure 8:**
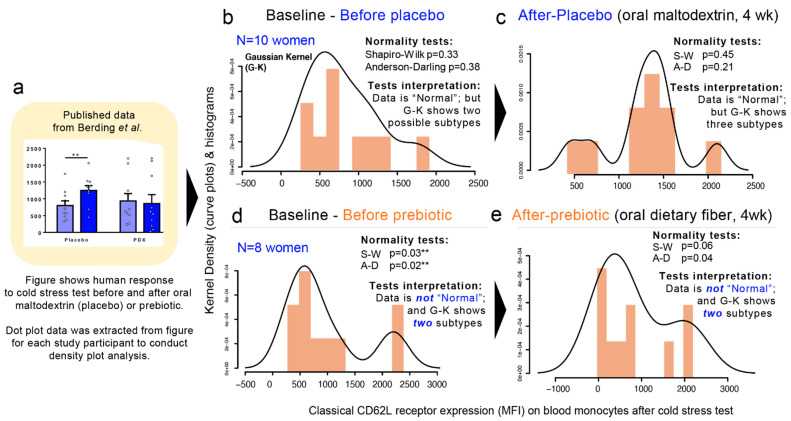
Example of multimodality in human outcome data. (**a**) Data was extrapolated from dot plot in study published by Berding et al. [87] (**b**,**c**) Outcome data for “placebo” maltodextrin group before and after cold stress test. (**d**,**e**) Outcome data for “prebiotic” fiber group before and after cold stress test. Panels B and D illustrate that two different groups of individuals sampled for the study have different degrees of susceptibility. Asterisks indicate significance *p* < 0.05.

To expand our analysis from that of microbiome hGM-FMT studies to that of other multifactorial diseases, we then examined published data from a double-blind, randomized, placebo-controlled, crossover design study in which the efficacy of a prebiotic dietary fiber (polydextrose) in improving cognitive performance an acute stress response in healthy individuals was investigated [87]. Using pre- and post-intervention data extrapolated from a figure in the publication, herein, we show that multimodality is present in human-derived outcomes. (Figure 8a–e). In the context of the simulations herein presented, this analysis represents the complexity of biomedical research and illustrate means to visualize disease subtypes. In this example, we show the “high”, “middle”, or “low” responders to cold stress regardless of the treatment (placebo vs. prebiotic fiber). Note that at baseline, two samples of individuals have two different distributions (normality tests and multimodality). In the figure, panels 8B and D illustrate that two different groups of individuals sampled for the study have different degrees of susceptibility. It is also important to note that at baseline, the two samples of individuals have two different distributions (normality tests and multimodality; Figure 8b,d). In conclusion, re-analysis of data from this human clinical trial that used maltodextrin as dietary placebo illustrates multimodal variability/differences in the stress responses between the two human groups and after the placebo consumption.

## 4. Discussion

Understanding dataset multimodality and identifying strategies to address such source of variability in statistics is an emerging field in applied statistics to help address the complexity of multipeak data sets to improve study inferences and reproducibility in various fields of science, including biomedical research. Despite the inclusion of large numbers of human subjects in microbiome studies, the causal role of the human microbiome in disease remains uncertain. Exemplifying that a large N is not necessarily informative with complex human diseases, a large metanalysis [88] of raw hGM data from obese and IBD patients showed that human disease phenotypes do not always yield reproducible interlaboratory predictive biological signatures. Even when hundreds of individuals are studied, especially, if the “effect size for the disease of interest” is narrow (i.e., in obesity; larger in IBD) relative to the variability of the disease. For the human IBD subtypes (i.e., ulcerative colitis and Crohn’s disease), the metanalysis [88] concluded that only the ileal form of Crohn’s disease showed consistent hGM signatures compared to both healthy control donors and patients with either colonic Crohn’s disease or ulcerative colitis [89], but no consistent signatures were observed for obesity.

Using a simple strategy of assuming random numbers drawn from an observed sample distribution, we have analytically illustrated that increasing N yields aberrant and/or conflicting statistical predictions, which depend on the patterns of disease variability and presence of disease subtypes (data modes). Specifically, our simulations revealed that the number of discernable data subtypes may wax and wane as N increases, and that increasing N does not uniformly enable the identification of statistical differences between groups. Furthermore, subjects randomly selected from a multimodal diseased population may create groups with statistical differences that do not always have the same direction. Especially, (i) if the human disease of interest exhibits variable phenotypes (e.g., cancer, obesity, or asthma) and (ii) if multivariable cage-clustered data analyses are not used to account for intraclass correlation coefficient of phenotypes within/between animal cages.

Under the “weak law of large numbers” principle in mathematics (Bernoulli’s theorem [73,74,75]; see references for further illustration [90]), as N increases, the distribution of the study/sample means approximates the mean of the actual population, which facilitates the identification of statistically significant (but not biologically meaningful) differences between otherwise overlapping sample datasets. Commonly used statistical methods (e.g., *t*-tests; parametric vs. nonparametric) are designed to quantify differences around the sample centers (mean, median) and range of dispersion (standard errors or deviation) of two groups. However, these methods do not account for the distribution shape (unimodal vs. bi/multimodal) of the compared datasets. With arbitrary increases in N, what is insignificant becomes significant, thus increasing the tendency for the null hypothesis to be rejected despite clinically subtle differences [91,92].

To guide the selection of sufficient N (cases) or disease data subtype, herein we highlight the use of two simple statistical steps: (i) to first determine if the shape of the dataset is unimodal (e.g., dip test), and if not unimodal, then (ii) to use statistical simulations and tests to determine the number of modes/data values of interest, and finally, to (iii) perform Monte Carlo simulations using the statistical analysis conducted by scientists on their experimental data to quantify the frequency by which random sampling could interfere with the *p*-value computed. Such forms of Monte Carlo adjusted *p*-values can easily be performed using GraphPad or similar software (R, STATA), which are widely used in the literature. Doing so facilitates the objective design of personalized/disease subtyping experiments. Although comparisons between group means is important because some diseases are truly different, findings from our own hGM-FMT study [52] and others [21,23] highlight the relevance of studying disease subtypes and the sources of variability by personalizing the functional analysis of the hGM in mice (i.e., that both “pathological” and “beneficial” effects can be seen in hGM-FMT mice independent of donor disease status). For example, in our own work, the functional characterization of “beneficial” or “nonbeneficial” disease microbiome subtypes in IBD patients at times of remission could lead to the identification of an ideal patient fecal sample for future autologous transplantation during times of active disease. Therefore, personalized research has the potential to identify different functional microbiome subtypes (on a given outcome, e.g., assay or hGM-FMT mice) for one individual. 

One pitfall of traditional statistics that are based either on mean and SD, or on nonparametric median and ranking methods, is that only central and dispersion parameters are used for analysis, which does not represent the data distribution shape. With mean and SD consistent with the observed data, there is no guarantee that the simulated data would match the whole distribution of the observed data.

With respect to determining unimodality, easily implementable tests to quantify data modality are available in STATA (statistical functions *diptest* and *mode;* proprietary and community contributed) and R (Package *multimode*, community contributed) [93]. The dip test [69] quantifies departures from unimodality and does not require a priori knowledge of potential multimodality, and thus, information can be easily interpreted from the test statistics and the *p*-value [83,94]. Although reports and comparative analysis of statistical performance have been described for various multimodality tests (e.g., dip test, bimodality test, Silverman’s test, and likelihood ratio test [95], and kernel methods), including simpler alternatives that use benchmarks to determine the influence of data outliers [78,79,83,96], it is important to emphasize that every method depends on its intended application and data set/shape [84], and thus must be accompanied by the inspection of the data distributions modes.

## 5. Conclusions

In conclusion, by conducting a series of simulations and a review of statistical methods in current hGM-FMT literature, we extensively illustrate the constraints of increasing N as a main solution to identify causal links between the hGM and disease. We also highlight the integral role of multivariable cage-clustered data analyses, as previously described by our group [8]. Herein, we provided a conceptual framework that integrates the dynamics of sample center means and range of dispersion from the compared datasets with kernel and violin plots to identify “data disease subtypes” to address sample size and data multimodality. Biological insights from well-controlled, analyzed, and personalized analyses will lead to precise “person-specific” principles of disease, or identification of anti-inflammatory hGM, that could explain clinical/treatment outcomes in patients with certain disease subtypes and self-correct, guide, and promote the personalized investigation of disease subtype mechanisms.

## Data Availability

All datasets analyzed for this report are included in the following published articles [21,51,52] and/or their supplementary information files.

## Supplementary Materials

The following are available online at https://www.mdpi.com/2075-4426/11/3/234/s1, Figure S1: Generation of random datasets of integer and decimal numbers using functions designed to draw numbers from a uniform and gaussian distribution, Figure S2: Further examples for data simulations with R2 value illustrate linearity as illustrated in Figure 1D, Figure S3: GraphPad Methods for Monte Carlo simulation, Figure S4: Monte Carlo Gaussian 100,000 simulations, Figure S5: Markov chain Monte Carlo (MCMC) simulations and examples of dip test, Figure S6: 16S microbiome profiles of hGM-SAMP fed an AD or AD-modified diet for 24 weeks, S7: Categorical data simulations and violin plots illustrate that categorical data exhibit multimodality and affects statistical reproducibility of random data. Table S1: Comparative percentages of simulations that yielded significant results for two statistical approaches, Video S1: Time-Lapse Examples doi:10.6084/m9.figshare.13377407 (https://figshare.com/s/dcf154ce73c5bc086e80).
