# Supplementary material for: ‘Statistical Irreproducibility’ Does Not Improve with Larger Sample Size: How to Quantify and Address Disease Data Multimodality in Human and Animal Research
Source: J Pers Med. 2021 Mar 23;11(3):234. doi: 10.3390/jpm11030234 (PMC8005169; doi:10.3390/jpm11030234)
Supplement: Supplementary file 1 [file jpm-11-00234-s001.pdf]

‘Statistical Irreproducibility’ Does Not Improve with Larger Sample Size: How to Quantify and Address Disease Data Multimodality in Human and Animal Research

Abigail R Basson[1,2], Fabio Cominelli[1,2,3,4], Alexander Rodriguez-Palacios*[1,2,3,4]

[1] Division of Gastroenterology & Liver Diseases, Case Western Reserve University School of Medicine, Cleveland, OH, USA.
[2] Digestive Health Research Institute, University Hospitals Cleveland Medical Center, Cleveland, OH, USA.
[3] Mouse Models Core, Silvio O’Conte Cleveland Digestive Diseases Research Core Center, Cleveland, OH, USA.
[4] Germ-free and Gut Microbiome Core, Digestive Health Research Institute, Case Western Reserve University, Cleveland, OH, USA.

Alex Rodriguez-Palacios (axr503@case.edu) [Corresponding Author]

Supplementary Materials

SECTION A: Supplementary Tables

Suppl. Table 1 Comparative percentages of simulations that yielded significant results for two statistical approaches

SECTION B: Supplementary Figures

Suppl. Fig. 1 Generation of random datasets of integer and decimal numbers using functions designed to draw numbers from a uniform and gaussian distribution

Suppl. Fig. 2 Further examples for data simulations with R^2 value illustrate linearity as illustrated in Figure 1D.

Suppl. Fig. 3 GraphPad Methods for Monte Carlo simulation

Suppl. Fig. 4 Monte Carlo Gaussian 100,000 simulations

Suppl. Fig. 5 Markov chain Monte Carlo (MCMC) simulations and examples of dip test.

Suppl. Fig. 6 16S microbiome profiles of hGM-SAMP fed an AD or AD-modified diet for 24 weeks.

Suppl. Fig. 7 Categorical data simulations and violin plots illustrate that categorical data exhibit multimodality and affects statistical reproducibility of random data.

Supplementary Table 1. Comparative percentages of simulations that yielded significant results for two statistical approaches

	Inverse Normal Gaussian	Monte Carlo Normal Gaussian	
Simulation, n= and statistical test	50 T-tests (significant cumulative linear pattern*) (95%CI=)	100,000 Adjusted T-tests (overall significance with N=63/group)(95%CI=)[a]	100,000 Adjusted One Way with multiple comparison Tukey test[b] (95%CI=)
Dis1 vs Dis2	35.3% (22.9, 50.8)	57.7% (57.4, 58.0)	37.8% (37.5, 38.1)
Dis1 vs Healthy	58.8% (43.2, 71.8)	9.1% (8.9, 9.3)	3.8% (3.7, 3.9)
Dis2 vs Healthy	ND	78.3% (78.0, 78.6)	59.6% (59.3, 59.9)
			One Way ANOVA
			$p<0.05$ 68.1% (68.4 to 67.8)
			$p>0.05$ 31.9% (32.2 to 31.6)

Table based on randomly simulated data derived from unimodal distributions.

*Not overall p-value at N=63. ND, not determined.

[a,b] Notice that the percentage of simulations achieving significance is inflated when analysis for three groups is conducted with T-tests (instead of ANOVA) which does not control for false positives due to family errors. Proper comparison between >2 groups should be performed with methods to control for such family errors (*e.g.*, ANOVA-post-hoc Tukey statistics). Note that the percentage is different as illustrated in Figure 1D because the patterns with non-linear behavior are not considered.

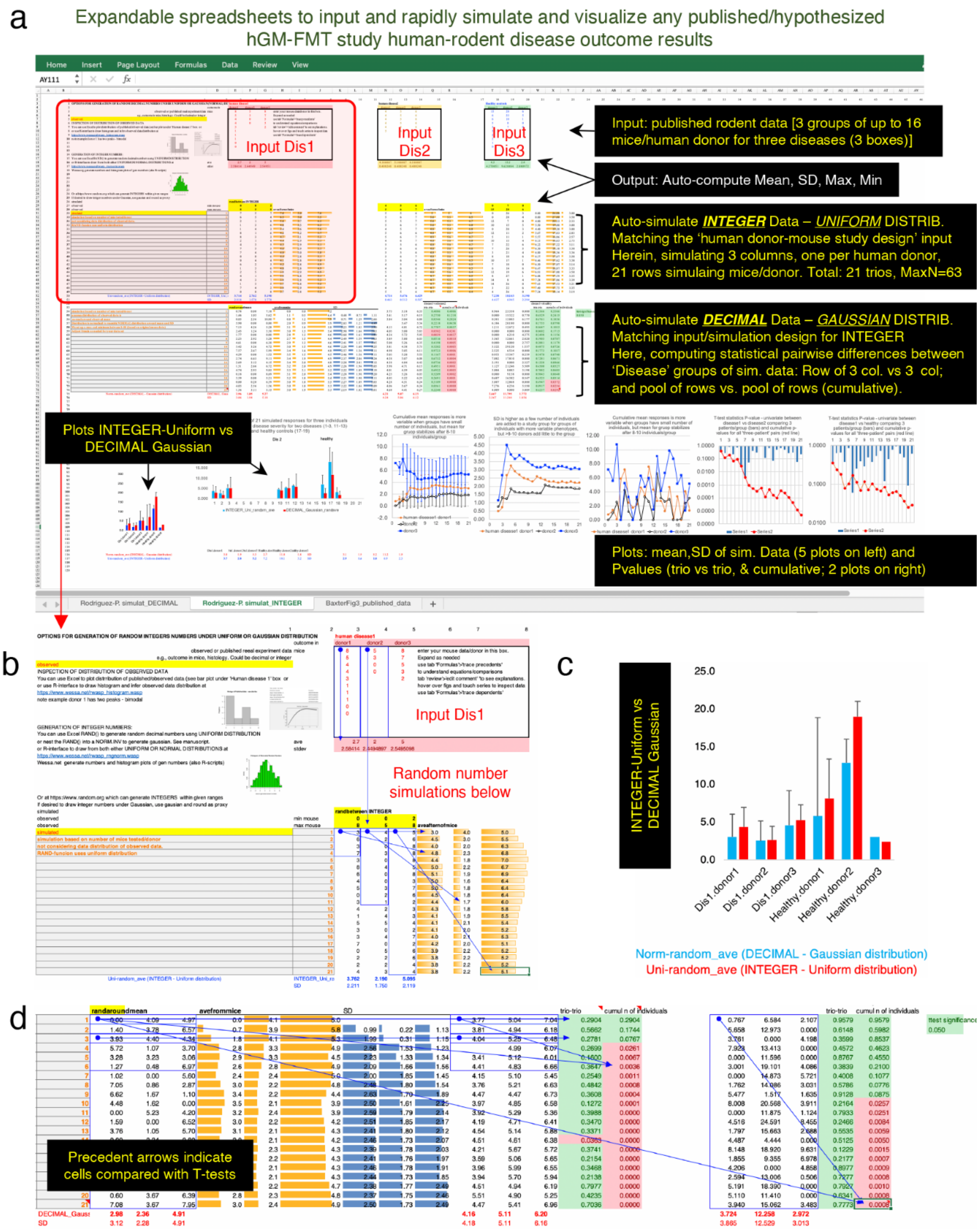

Supplementary Figure 1. Generation of random datasets of integer and decimal numbers using functions designed to draw numbers from a uniform and gaussian distribution. a) Input and output mean used to simulate integer and decimal data with corresponding plots and p-values. **b)** Inspection of distribution of observed data shows that data has uniform distribution. **c)** Bar plot illustrates similarity between random generated integer- uniform and decimal-gaussian distribution. **d)** T-test comparison for trio-trio and cumulative N of individuals.

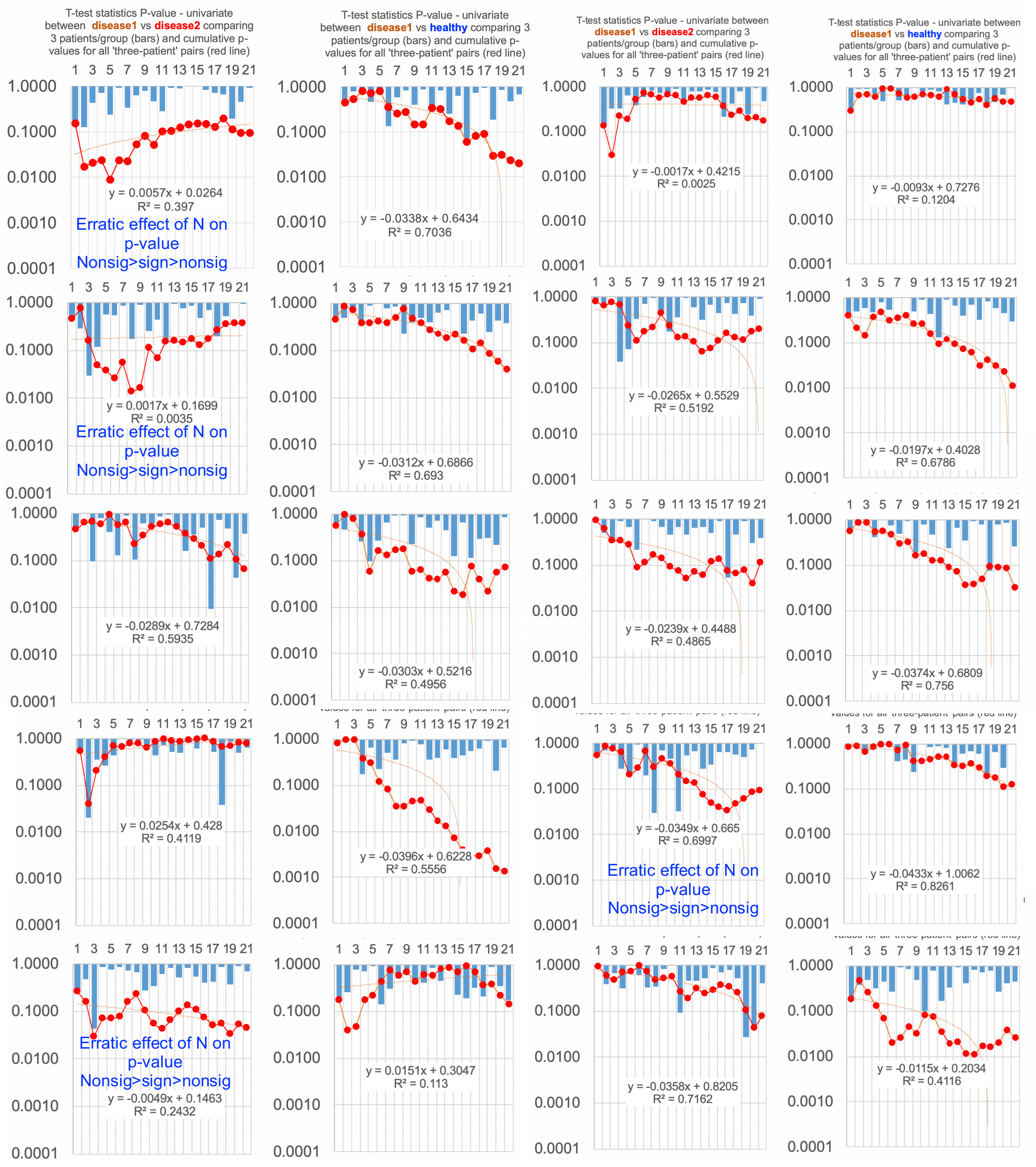

Supplementary Figure 2. Further examples for data simulations with R^2 value illustrate linearity as illustrated in Figure 1D. Computed R^2 value (mean 0.51±0.23, 20 simulations) illustrate the linearity of the correlation between N and statistical significance. Y axis, p-value of the differences using 2-group Student-t test.

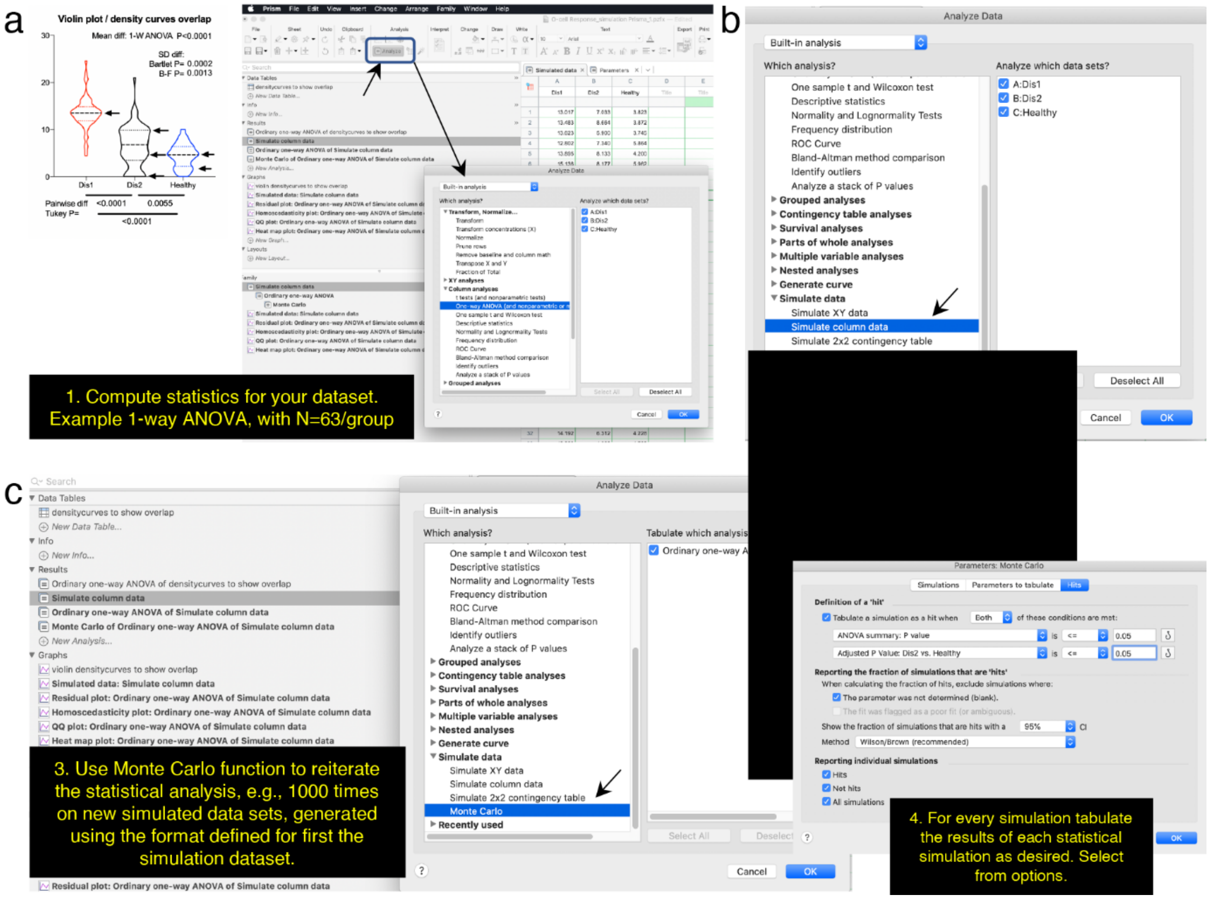

Supplementary Figure 3 GraphPad methods for Monte-Carlo simulations with gaussian distribution. See Supplementary File 2 for associated dataset.

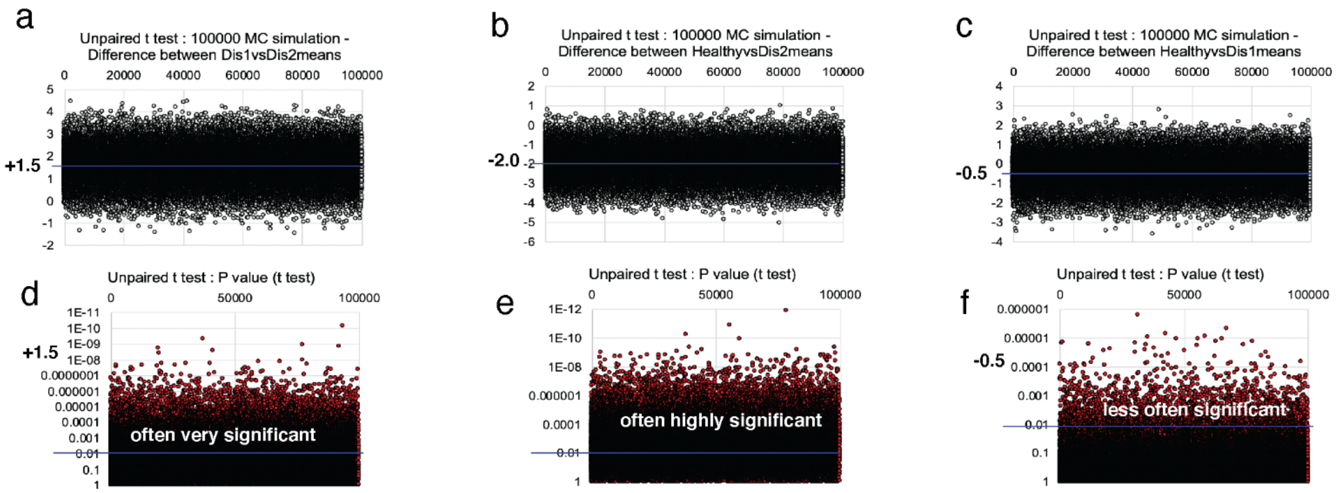

Supplementary Figure 4. Monte Carlo Gaussian 100,000 simulations. Simulations performed in R software. **a, b & c)** are group mean differences. **d, e & f)** illustrate p-values of mean group differences.

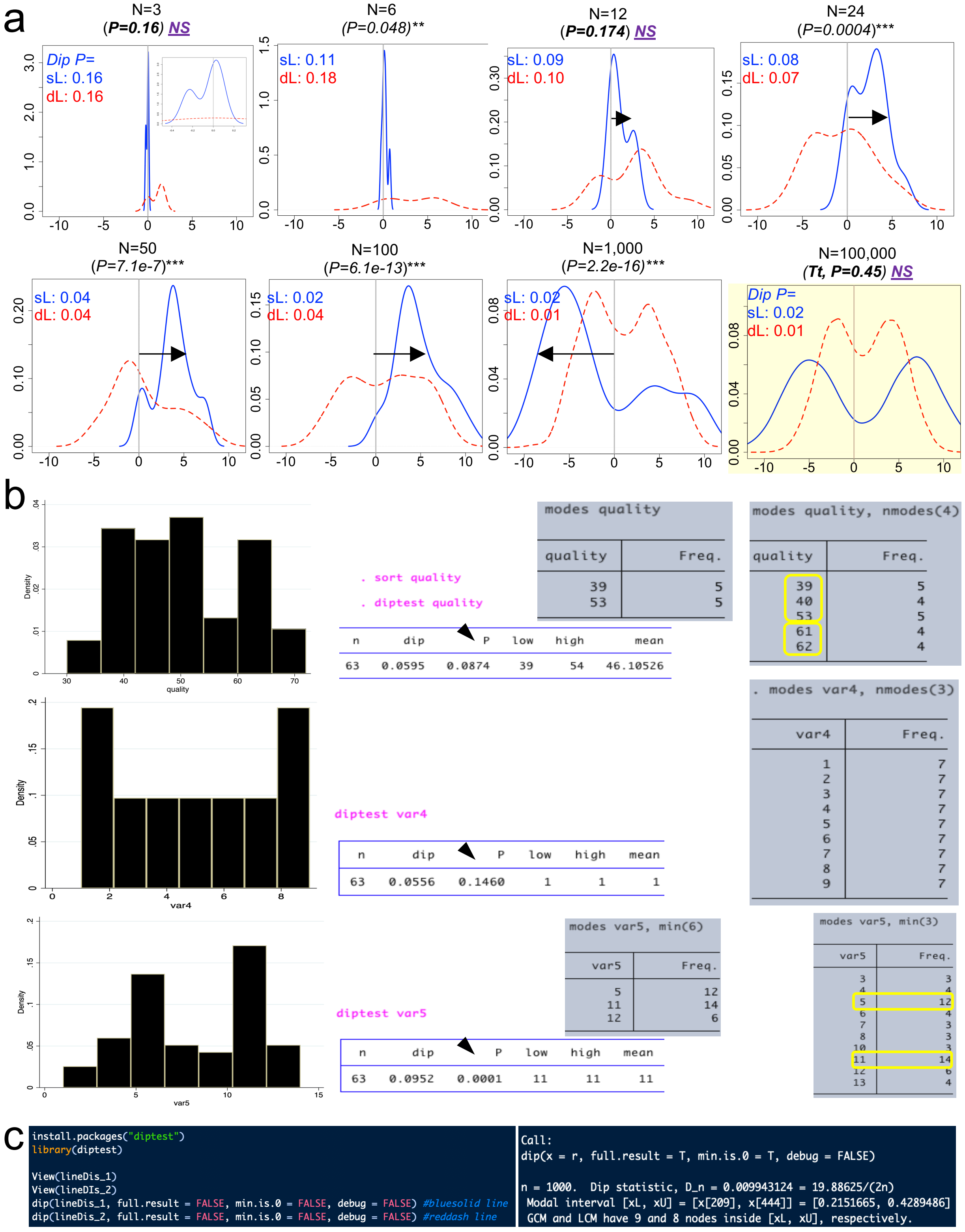

Supplementary Figure 5. Markov chain Monte Carlo (MCMC) simulations and examples of dip test. Random walk Markov chain Metropolis-Hastings' algorithm to simulate random sampling accounting for the hypothetical dependence of two different disease subtypes (complement to plots presented in Figure 5).

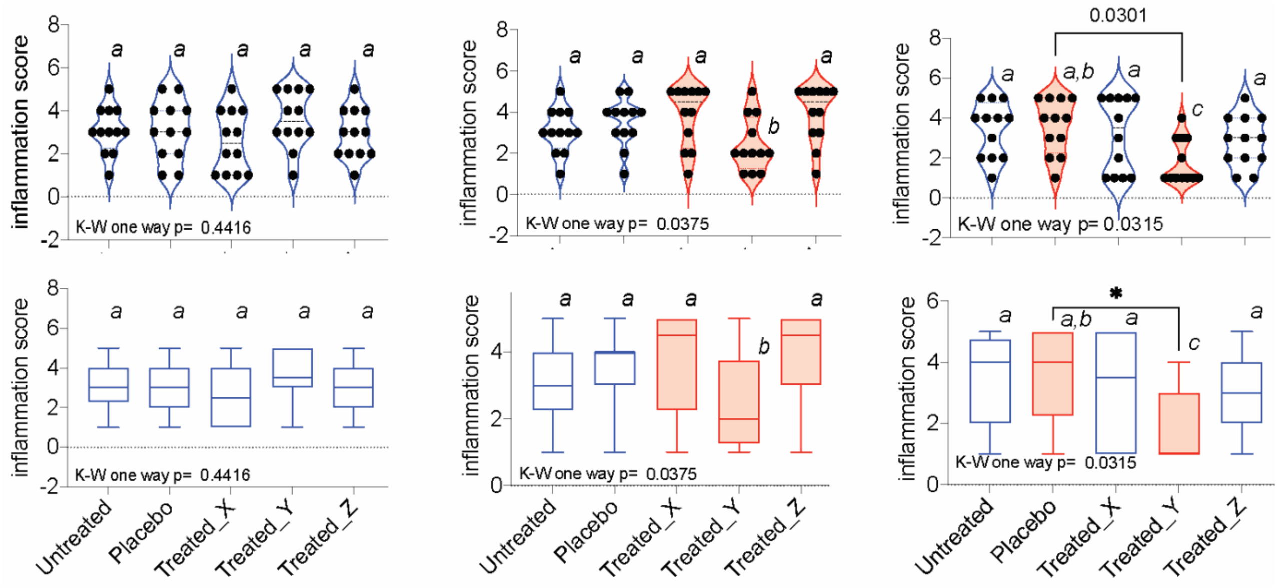

Supplementary Figure 6. Categorical data simulations and box plots illustrate multimodality principles and effect on statistical reproducibility of random data. Example of box plots to illustrate corresponding comparisons (see Figure 6).

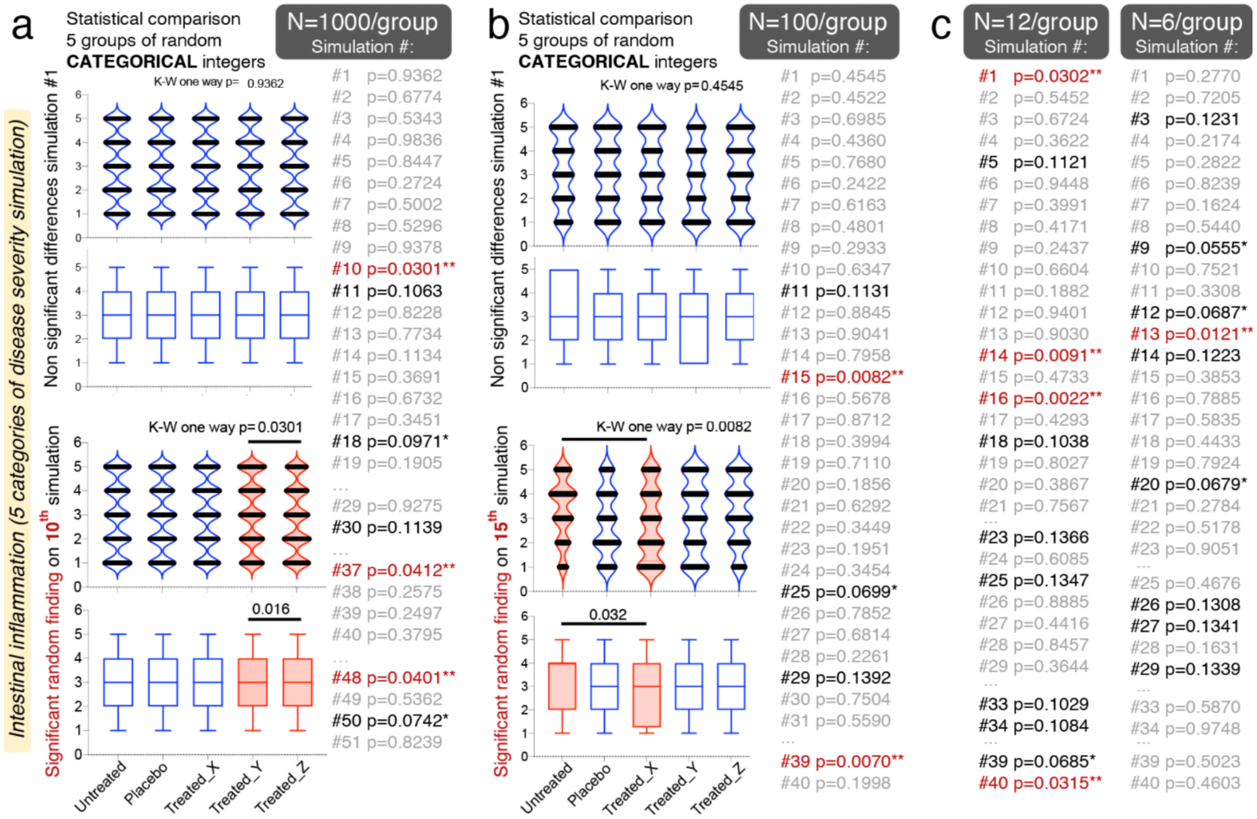

Supplementary Figure 7. Categorical data simulations and violin plots illustrate that categorical data exhibit multimodality and affects statistical reproducibility of random data. a) Example of violin plot and its correspondent box plot for non-significant (expected similarity across treatment groups, p>0.05) and false irreproducible non-significant statistical differences (p<0.05) across comparisons for 5-groups. Random simulations with integer datasets and listed one-way K-W p-values for N=1000/group. Note multimodal curve shape in violin plots. b) Same as panel 6a, for N=100/group. c) Lists of p-values for N=12 and N=6/group. Black font in p-values if <0.15.
